# The Influence of Preoperative Symptoms on the Death of Patients with Small Intestinal Neuroendocrine Tumors

**DOI:** 10.1245/s10434-016-5703-4

**Published:** 2016-11-30

**Authors:** John Eriksson, Hans Garmo, Per Hellman, Catharina Ihre-Lundgren

**Affiliations:** 1 Department of Surgical Sciences, Faculty of Medicine, Uppsala University, Uppsala, Sweden; 2 Division of Cancer Studies, Cancer Epidemiology Group, Research Oncology, King’s College London, UK/Regional Oncologic Center, Uppsala University, Uppsala, Sweden; 3 Department of Molecular Medicine and Surgery, Karolinska Institute, Stockholm, Sweden

## Abstract

**Background:**

Small intestinal neuroendocrine tumors (SI-NETs) are uncommon tumors with an annual incidence of about 1 per 100,000. Usually, SI-NETs have a slow progression, and patients often present with generalized disease. Many patients do well, and the disease has a relatively favorable 5-year survival rate. Some SI-NETs, however, have a more negative prognosis. This study aimed to establish prognostic factors for death identifiable at primary surgery.

**Methods:**

A nested case-control study investigated 1150 patients from the cohort of all patients with a diagnosis of SI-NETs in Sweden between 1961 and 2001. The study cases consisted of all patients who died of SI-NETs during the study period. Each case was assigned a control subject matched by age at diagnosis and calendar period. Possible prognostic factors [gender, degree of symptoms, indication for surgery, World Health Organization (WHO) stage] were evaluated in uni- and multivariable analyses.

**Results:**

The patients with symptomatic disease had an increased risk of dying. The indication for primary surgery influenced survival, showing a more negative prognosis for elective surgery. The WHO stage influenced survival, and stage 4 patients had an almost threefold risk of dying compared with stages 1 to 3b patients.

**Conclusions:**

This study showed that preoperative symptoms are important in prognostication for SI-NETs. Hormonal symptoms generally signify a patient with a more advanced disease stage and a worse prognosis. Including symptomatic disease together with the WHO stage and grade could possibly increase the accuracy of prognostication.

Small intestinal neuroendocrine tumors (SI-NETs) are rare tumors with an annual incidence of about 1 per 100,000 and an increasing annual incidence during the last three decades.^1^–^3^ Despite their rarity, SI-NETs are the most common of the gastrointestinal NETs, accounting for 42% of all gastrointestinal NETs. They also are the most frequent tumors originating in the small intestine, most frequently occurring in the ileum.^2^

Usually, SI-NETs have an indolent course, with few and nonspecific primary symptoms initially. Some patients have mild symptoms for years, or even decades, before diagnosis.^4^ Although the majority of patients present with disease that has already spread (50–70% lymph node metastases, 25–50% distant metastases^2^,^5^,^6^), the prognosis often is relatively favorable, with an overall 5-year survival of 60 to 70 %.^1^,^2^,^5^,^7^,^8^

The clinical syndrome of SI-NET (flushing, diarrhea, occasional pulmonary obstruction, and in severe cases, right-sided heart failure) is rarely present before liver metastases have occurred.^9^ The hormonal symptoms are related to bioactive peptides produced by the tumor cells (e.g., serotonin and tachykinins) metabolized by the liver in localized disease.^10^,^11^ Besides circulatory and obstructive phenomena, the released peptides also may cause a fibrotic reaction by stimulating fibroblasts to collagen production. This may have a local effect in the vicinity of the metastases or may systemically cause stenosis or insufficiency of the tricuspid and pulmonary valves, resulting in the right-sided heart failure seen in advanced carcinoid syndrome.

Prognostication for the individual patient is problematic. Seemingly similar tumors have a heterogeneous behavior for reasons not completely understood. The World Health Organization (WHO) staging and grading system has improved the accuracy of prognostication,^12^,^13^ but a considerable interquartile variation still exists.^14^ Currently, the best prognostic indicators are invasive growth, tumor burden, and metastatic disease.^7^,^13^,^15^–^17^

This study aimed to establish prognostic factors for death of patients with SI-NETs that are identifiable preoperatively at the time of the first surgery. Our main hypothesis was that patients with hormonal symptoms at the time of surgery have a worse prognosis.

## Materials and Methods

### Patient Identification

With support from a local ethics committee (UPS02-066), we identified all patients registered in the Swedish Cancer Registry (SCR) between 1961 and 2001 as having SI-NET (3740 patients). The SCR, established in 1958, requires clinicians and cytologists/pathologists to report diagnosed tumors. The registry is indexed on a unique national registration number given at birth. National coverage of the SCR was 95.5% in 1978^18^ and 96.7% in 1998.^19^ The coverage currently is considered to be nearly 100%.^20^ The Swedish Cause of Death Registry (SCDR) registers the most likely cause of death according to the clinician issuing the cause of death certificate.

Using the design of a nested case-control study, a cross-match between the SCR and the SCDR was performed, identifying all patients with an SI-NET as their primary cause of death (809 patients). Review of the registry found 443 patients with the cause of death likely attributable to SI-NETs but not defined as death due to SI-NET in the SCDR. Given that these patients had a known SI-NET, they also were included for further analysis.

**Fig. 1:**
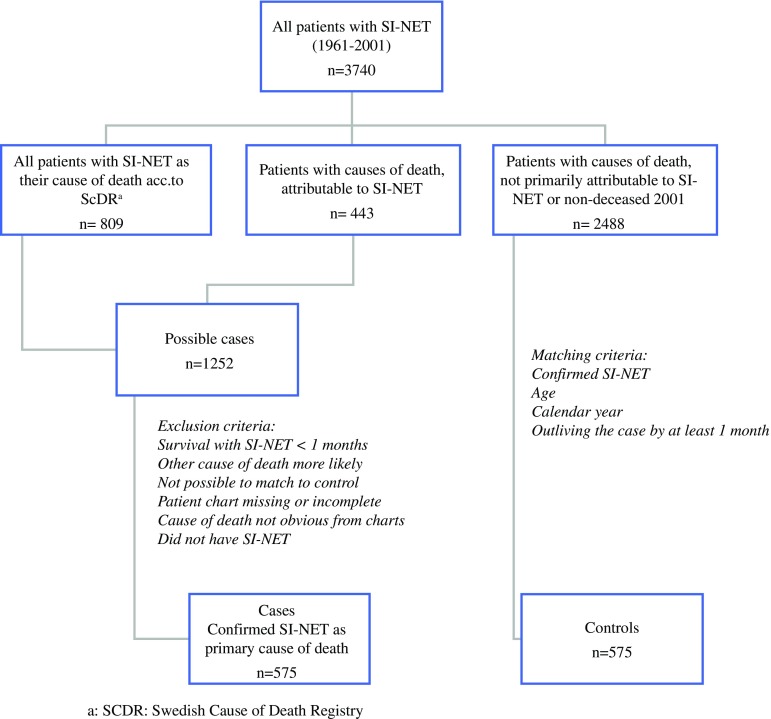
Matching process flow chart

At review, patients were excluded when the cause of death was deemed not be due to SI-NET. We omitted anyone who had lived less than 1 month with the diagnosis of SI-NET and those with SI-NETs found at autopsy. This exclusion was performed to eliminate the influence of perioperative complications and preoperative factors not exclusive to an SI-NET. The hospital records of the remaining patients were reviewed, and only patients confirmed as likely to have died of their SI-NET were accepted as study cases (575 cases). These cases were matched to control subjects outliving the case from the cohort (Fig. 1).

The cases and control subjects were matched by calendar year of diagnosis and age at diagnosis. To avoid overmatching, gender was not used as a matching variable. In summary, the cases were patients who had died due to SI-NET, and the control subjects were patients of about the same age as the case patient whose diagnosis occurred within the same period and who outlived the case by at least 1 month.

Clinical records, laboratory protocols, and histopathology reports were requested for all the patients included in the study. Data were reviewed and recorded in a protocol in Filemaker (Filemaker Pro; Filemaker Inc., Santa Clara, California).

### Symptom Scoring

We adapted the carcinoid symptom severity scale (SSS), used previously^21^ and described by Schell et al.,^10^,^11^ to evaluate the degree of carcinoid syndrome the patient was experiencing (Table 1).We adjusted the scale because scores 2 and 3 showed no apparent difference with regard to symptom frequency (1–4 times daily vs. 5–7 times weekly). The difference in Wessels’ score between 2 and 3 is primarily associated with the patient’s reported lifestyle effects. We found this difference difficult to ascertain from patient records and therefore a possibility of bias. Our adapted score is therefore more focused on the frequency of symptoms.

**Table 1:** Symptom severity scale, adapted from Wessels et al.^10^,^11^

Score	Description	Symptoms	Frequency	Lifestyle effects
1	No symptoms	None	0	None
2	Mild Symptoms	Diarrhea, flushing, or wheezing	1–4 times daily	None to minimal
3	Symptoms impacting daily living	Diarrhea, flushing, or wheezing	5–7 times daily	Restricts patient from leaving home for prolonged periods
4	Severe symptoms	Diarrhea, flushing, or wheezing	Multiple daily episodes (>7)	Symptoms require significant reorganization of daily activities to accommodate them; patients rarely leave home, must be close to bathroom facilities and medical supplies
5	Disabling symptoms	Diarrhea, flushing, and wheezing	Multiple daily episodes	Symptoms are disabling; patients are unable to leave home or require hospitalization

### Cause of Surgery

Surgery in this study referred to the first surgery the patient underwent at the time of diagnosis. If the surgery was exploratory or if the intended surgical procedure was aborted and a resection with curative intent followed within 3 months, these were defined as a single procedure.

The causes of surgery were grouped into five categories:

*Emergency surgery* any emergency abdominal operation in which the diagnosis of SI-NET was not known preoperatively

*Elective surgery* planned surgery for a known SI-NET

*En passant surgery* tumor found during a procedure intended for another abdominal disorder

*Explorative surgery* a non-emergency surgery because of a suspected intraabdominal disease or because of a palpable mass, in which no preoperative diagnosis was set

*No surgery*

### Bowel Obstruction

In the group of patients with SI-NET diagnosed through an emergency procedure, we scrutinized the indication for their laparotomy and divided them into the following two categories: surgery due to signs of bowel obstruction and emergency surgery for other reasons.

### WHO Stage and Grade

From our data, patients were grouped into tumor-node-metastasis (TNM) stages, as proposed by Rindi et al.^10^ The staging was determined on the basis of perioperative data from the operative report. Only six patients in this study had a recorded postoperative Ki-67 value, making it impossible to analyze its implications or to establish a WHO grade.

Data were analyzed using RStudio.^22^ Conditional logistic regression was used to estimate odds ratios (OR) of death from the SI-NET, with 95% confidence intervals. An association was considered statistically significant when the 95% confidence interval for the OR did not include 1. We also computed multivariable analyses, in which groups of recorded items included the symptom severity score (SSS), type of surgery, gender, and WHO stage.

### Carcinoid Heart Disease

Patients who had undergone a preoperative ultrasonography of the heart with a recorded insufficiency of the tricuspid valve were defined as having a carcinoid heart disease.

## Results

Both the cases and the control subjects in our groups had a predominance of women, similar to the incidence reported in the surveillance, epidemiology and end results (SEER) database (men, 47.4%; women, 52.6%).^9^ The mean age at diagnosis was similar in the two groups, with a general mean age of 66.9 ± 10.30 years. Gender was not found to be a prognostic factor for the death of patients with SI-NETs.

### Preoperative Symptoms

The presence of carcinoid symptoms at the time of primary surgery, defined in this report as an SSS greater than 1, was found to be a prognostic factor for death of SI-NET patients. Our data also indicated that the degree of symptoms correlates with prognosis (Table 2). Symptomatic patients generally had a more advanced disease, as evidenced by a higher WHO stage. Of all the patients without hormonal symptoms whose WHO stage was known, 62% had stages 1–3b disease, whereas 66% of the symptomatic patients had stage 4 disease (data not shown). The patients with acute abdominal symptoms generally did not have hormonal symptoms. Hormonal symptoms were twice as common among the patients who underwent surgery in the nonemergency setting (35 vs. 18%).

**Table 2:** Patient data

	Cases		Controls		Total		OR UV		OR MV	
Patient data	*n*	%	*n*	%	*n*	%	OR UV	95% CI	OR MV^a^	CI MV
Total	575	100	575	100	1150	100				
Males	270	47.0	246	42.8	516	44.9	1.00	Reference	1.00	Reference
Females	305	53.0	329	57.2	634	55.1	0.85	0.68–1.07	0.86	0.55–1.36
Symptom Severity Score^b^										
1	318	55.3	394	68.5	712	61.9	1.00	Reference	1.00	Reference
2	140	24.3	77	13.4	217	18.9	2.28	1.64–3.15	1.89	1.32–2.70
3	56	9.7	29	5.0	85	7.4	2.52	1.54–4.10	2.01	1.17–3.47
4–5^c^	24	4.2	7	1.2	31	2.7	4.17	1.76–9.87	3.59	1.41–9.17
Data not available	37	6.4	68	11.8	105	9.1	2.28	1.64–3.15	1.89	1.32–2.70
Operation type										
Elective	99	17.2	51	8.9	150	13.0	1.00	Reference	1.00	Reference
Emergency	194	33.7	215	37.4	409	35.6	0.46	0.31–0.69	1.14	0.62–2.07
Explorative	192	33.4	199	34.6	391	34.0	0.49	0.33–0.74	0.76	0.47–1.24
En passant	35	6.1	46	8.0	81	7.0	0.37	0.21–0.66	0.82	0.42–1.60
No surgery	12	2.1	2	0.3	14	1.2	2.68	0.57–12.57	8.04	1.48–43.68
Bowel obstruction										
No	443	77.0	435	75.7	878	76.3	1.00	Reference	Not included	
Yes	132	23.0	140	24.3	272	23.7	0.92	0.70–1.22		
WHO stage										
1–3B	158	27.5	290	50.4	448	39.0	1.00	Reference	1.00	Reference
4	263	45.7	136	23.7	399	34.7	3.61	2.65–4.90	1.99	0.83–4.76
Data not available	154	26.8	149	25.9	303	26.3	1.81	1.33–2.47	0.86	0.55–1.36
Carcinoid heart disease										
Yes	15	2.6	8	1.4	23	2.0	0.62	0.17–2.26	Not included	
No	22	3.8	6	1.0	28	2.4	1.00	Reference		
Data not available	538	93.6	561	97.6	1099	95.6	0.26	0.11–0.65		

*OR* odds ratio, *UV* univariable, *CI* confidence interval, *MV* multivariable, *SSS* symptom severity score

^a^ Variables included in the multivariable analysis were gender, SSS, type of surgery, World Health Organization (WHO) stage and age

^b^ Adapted SSS as described by Wessels et al.^11^

^c^ SSS 4 and 5 were grouped together because of scarcity of patients with severe symptoms

Because of this finding, we computed a univariable analysis, grouping patients into either symptomatic (*SSS* > 1) or nonsymptomatic (*SSS* = 1) patients and into WHO stages (Table 3). In this analysis, more than half of the control subjects were nonsymptomatic patients without generalized disease, whereas the study patients were more likely to be symptomatic and to have a more advanced disease stage. The patients with hormone-related symptoms had more than double the risk for dying of SI-NETs in both instances compared with the patients in the same stage of disease but with no signs of hormone-related symptoms.

**Table 3:** Hormone-related symptoms and WHO stage

	Cases		Controls		Total		OR UV	
	*n*	%	*n*	%	*n*	%	OR UV	95% CI
Symptoms and WHO stage								
Total	575	100	575	100	1150	100		
SSS^a^ 1 and WHO 1–3b	117	20.3	229	39.8	346	30.1	1.00	Ref.
SSS >1 and WHO 1–3b	39	6.8	53	9.2	92	8.0	1.48	0.92–2.39
SSS 1 and WHO stage 4	127	22.1	84	14.6	211	18.3	3.00	2.06–4.36
SSS >1 and WHO stage 4	133	23.1	46	8.0	179	15.6	6.06	3.90–9.42
Missing data	159	27.7	163	28.3	322	28.0	1.92	1.37–2.68

*WHO* World Health Organization, *OR* odds ratio, *UV* univariable, *CI* confidence interval, *SSS* symptom severity score

^a^Adapted SSS

### Indication for the Primary Laparotomy

**Fig. 2:**
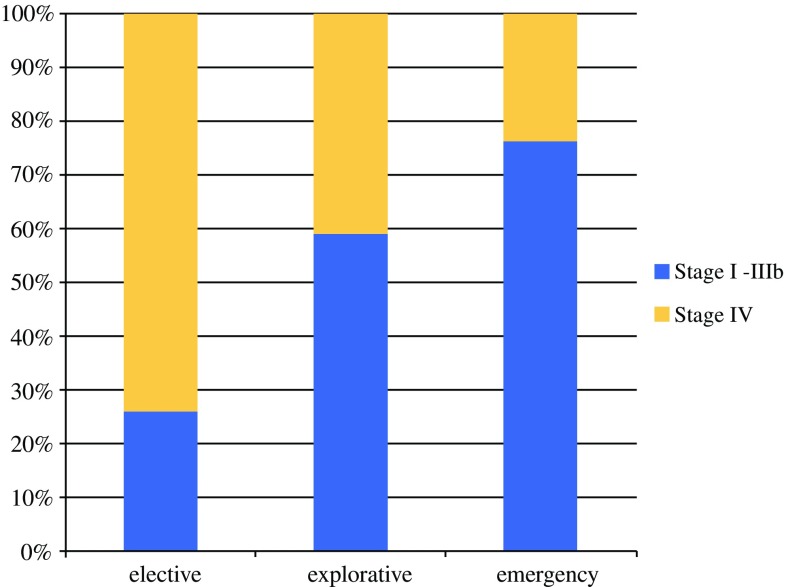
Type of surgery and WHO stage

In our material, if the primary surgery for SI-NET is acute or explorative, the prognosis is better in the acute setting (OR, 0.46 and 0.49, respectively; Table 2). However, this could not be shown in the multivariable analyses. Elective patients were more likely to have a more advanced disease. The findings showed that 76% of the patients undergoing surgery in the emergency setting had stages 1 to 3b disease, compared with 74% for the patients with stage 4 disease in the elective group (Fig. 2). Emergency surgery due to signs of bowel obstruction was not shown to be a prognostic factor for the death of SI-NET patients.

### WHO Stage

Almost all our patients (90%) were in one of two disease stages: stage 3B or 4. This unfortunately made it impossible to validate the WHO staging system from this material. However, we were able to show that stage 4 disease has a negative prognostic value relative to all other stages (Table 2).

### Carcinoid Heart Disease

Only 4% of our patients underwent a preoperative cardiac ultrasound, and 45% of these patients had some degree of tricuspid valve insufficiency. The analysis of tricuspid valve insufficiency as a risk factor for death among SI-NET patients was inconclusive and therefore not included in the multivariable analysis.

## Discussion

The presence of preoperative symptoms is a strong indicator of decreased survival for SI-NET patients, and the risk for dying of SI-NET increases with increased symptoms. To the best of our knowledge, ours was the most complete study and one of the largest population-based studies of SI-NET tumors to date. It included all patients with a confirmed death due to a SI-NET in Sweden during a 40-year period and represents about one third of all SI-NETs diagnosed in Sweden during the study period. Moreover, the nested case-control method used is one of the most sensitive techniques for evaluating retrospective materials.

Our data lacked preoperative 5-hydroxyindoleacetic acid (5-HIAA) and CgA values, making the assumption that increased symptoms actually are related to an increased amount of systemic hormonally active peptides difficult. Only 13% of the patients had a recorded preoperative 5-HIAA measurement, and CgA values were available only in the later years of this study. Of these patients, 73% were symptomatic, suggesting that symptomatic patients more often have increased biomarkers. However, due to the lack of more comprehensive data, biochemical data were not analyzed further. Symptomatic patients in this study did, however, have a more advanced stage of disease, making this assumption likely (i.e., a larger tumor load produces more hormonally active peptides). Usually, symptomatic patients have elevated CgA levels, which is an established prognostic factor.^14^

Additionally, in a previous study,^21^ we showed that the SSS increases with elevated 5-HIAA and CgA levels. For some patients, an increased frequency of diarrhea may be explained by extensive mesenterial fibrosis resulting in an impediment of the venous circulation and sub-obstructive symptoms. Nonetheless, a patient with an SSS higher than one will have a less favorable prognosis regardless of the cause. This finding was evident in both the uni- and multivariable analyses. However, scoring the level of symptoms retrospectively involves some issues. Different patients have seen different physicians with a range of experience from junior residents to senior endocrine surgeons. They have been seen in either the elective or the emergency setting, in which hormone-related symptoms may sometimes be overlooked. In the emergency setting, if there was no record pertaining to symptoms, the SSS was scored from subsequent entries in the chart whenever the patient’s preoperative symptoms were discussed. This added the risk of recall bias.

Of all the patients, only 44 (3.8%) had been given a preoperative somatostatin analog, and 18 (1.5%) had begun treatment with interferon before their first surgery for SI-NET. Because 96% of the patients were not receiving any therapy, this study reflects how symptoms correlate with prognosis unbiased by medical therapy.

The cause of primary surgery has previously not been studied in detail in relation to prognostication. Our findings demonstrated that the indication for surgery has prognostic value. A patient undergoing an emergency laparotomy for an unknown SI-NET generally will do better than a patient undergoing elective surgery. Therefore, symptoms of acute abdominal disease do not imply a dismal prognosis for the patient with a non-diagnosed SI-NET, and in our study, actually correlated inversely with the extent of disease.

We were unable to show that small bowel obstruction is a prognostic factor in SI-NET. This adds to the current recommendation that in case of emergency symptoms, the surgeon should continue with surgery also in cases with obvious disease spread, which answers a question sometimes raised clinically.

Unfortunately, only a few of our patients had undergone a preoperative cardiac ultrasound. Symptomatic patients would be expected to have signs of tricuspid valve insufficiency more often than nonsymptomatic patients. The absence of data, however, makes this assumption impossible to prove. It is not surprising that cardiac ultrasounds were not performed in the emergency setting but somewhat surprising that they were equally sparse in the elective group.

When encountering a patient with a newly diagnosed SI-NET, prognostication is difficult. Many patients do well with symptoms efficiently treated. Additionally, with increased knowledge and the introduction of somatostatin analogs and interferon treatments, as well as recent medical treatments (everolimus, sunitinib), improvements in surgical techniques, additions of several treatments for generalized liver disease (ablations, liver embolization), and possibly the recent peptide-receptor radiation therapy, SI-NET survival has improved during recent decades in Sweden.^13^,^23^,^24^ These results conflict with results from the SEER database, which show no improvement in survival during the same period.^16^ Indeed, the SEER database is incomplete and, for example, does not contain all the patients with SI-NETs found during emergency laparotomy. In some series, these encompass up to 30 to 40% of all SI-NETs^5^ (35% in our material).

Because an emergency operation was a positive prognostic predictor in our series, survival analysis using the SEER database seemed to be negatively biased. Nevertheless, prognostication remains a challenge, as evidenced by the large interquartile variation in survival for patients with the same WHO stage and grade in the SEER database.^3^ We note that we adjusted for the improved surgical techniques and more sophisticated medical therapies administered in the later years of this study by matching the cases with the control subjects according to time period. Given our data, we propose including the presence or absence of symptomatic disease in the staging system to increase its accuracy.

Most studies investigating SI-NETs have been based on single-center outcomes and are retrospective. In our material, we included all the patients with disease diagnosed and treated in Sweden who had an SI-NET exclusively as their primary cause of death. We excluded all other NETs and created a multicenter study, eliminating at least some of the aforementioned bias. We believe this is the strongest possible way to perform a retrospective study of prognostic factors for SI-NETs.

This study suggests that our treatment and surveillance algorithms should be more skewed toward increased vigilance and possibly more aggressive treatment for the hormonally symptomatic patient because these patients are the most likely to die of their SI-NET.
